# Dimerization with Cannabinoid Receptors Allosterically Modulates Delta Opioid Receptor Activity during Neuropathic Pain

**DOI:** 10.1371/journal.pone.0049789

**Published:** 2012-12-14

**Authors:** Ittai Bushlin, Achla Gupta, Steven D. Stockton, Lydia K. Miller, Lakshmi A. Devi

**Affiliations:** 1 Department of Pharmacology and Systems Therapeutics, Mount Sinai School of Medicine, New York, New York, United States of America; 2 Department of Neuroscience and Friedman Brain Institute, Mount Sinai School of Medicine, New York, New York, United States of America; UAE University, United Arab Emirates

## Abstract

The diversity of receptor signaling is increased by receptor heteromerization leading to dynamic regulation of receptor function. While a number of studies have demonstrated that family A G-protein-coupled receptors are capable of forming heteromers *in vitro*, the role of these heteromers in normal physiology and disease has been poorly explored. In this study, direct interactions between CB_1_ cannabinoid and delta opioid receptors in the brain were examined. Additionally, regulation of heteromer levels and signaling in a rodent model of neuropathic pain was explored. First we examined changes in the expression, function and interaction of these receptors in the cerebral cortex of rats with a peripheral nerve lesion that resulted in neuropathic pain. We found that, following the peripheral nerve lesion, the expression of both cannabinoid type 1 receptor (CB_1_R) and the delta opioid receptor (DOR) are increased in select brain regions. Concomitantly, an increase in CB_1_R activity and decrease in DOR activity was observed. We hypothesize that this decrease in DOR activity could be due to heteromeric interactions between these two receptors. Using a CB_1_R-DOR heteromer-specific antibody, we found increased levels of CB_1_R-DOR heteromer protein in the cortex of neuropathic animals. We subsequently examined the functionality of these heteromers by testing whether low, non-signaling doses of CB_1_R ligands influenced DOR signaling in the cortex. We found that, in cortical membranes from animals that experienced neuropathic pain, non-signaling doses of CB_1_R ligands significantly enhanced DOR activity. Moreover, this activity is selectively blocked by a heteromer-specific antibody. Together, these results demonstrate an important role for CB_1_R-DOR heteromers in altered cortical function of DOR during neuropathic pain. Moreover, they suggest the possibility that a novel heteromer-directed therapeutic strategy for enhancing DOR activity, could potentially be employed to reduce anxiety associated with chronic pain.

## Introduction

Among the many current targets being explored for the treatment of neuropathic pain are opioid and cannabinoid receptors [1], [2], which share similar signaling properties and are widely co-distributed in regions of the peripheral and central nervous system associated with ascending pain sensation, descending inhibition of pain, as well as emotional processing [3]–[10]. Activation of both receptor types can produce analgesia in humans and animals experiencing neuropathic pain [5], [11], [12] by a variety of mechanisms, including but not limited to, agonist stimulated induction of gene expression, receptor cross activation, cannabinoid induced release of opioid peptides and opioid induced release of endocannabinoids [13]–[15] . Opioid and cannabinoid receptor activation can also reduce anxiety and depressive-like phenomena, particularly through selective activation of subtypes of these classes of receptors, including the delta opioid receptor (DOR) subtype [16], [17] or cannabinoid type 1 receptor (CB_1_R) subtype [5], [18]–[20]. Previous studies have shown indirect inhibitory interactions between these two receptors. For example, DOR activity is increased in the brains of CB_1_R −/− mice [21], while CB_1_R activity is increased in the brains of DOR −/− mice [22], [23], indicating that DOR can modulate CB_1_R activity and vice versa. Additionally, anxiolytic-like responses induced by Δ^9^THC, a CB_1_R agonist, could be blocked by naltrindole, a DOR antagonist [24], suggesting that supraspinal interactions between DOR and CB_1_R can modify behavior.

G protein-coupled receptor heteromerization has been previously shown to enhance the repertoire of receptor signaling, thereby dynamically modulating receptor function [25], [26]. A recent set of studies examined direct interactions between CB_1_R and DOR in heterologous systems [22]. Co-expression of tagged DOR and CB_1_R leads to an increase in BRET signal, whereas co-expression of CCR5 and CB_1_R does not [27], indicating that DOR and CB_1_R are in close proximity to each other in cells expressing both receptors. Moreover, DOR and CB_1_R can be co-immunoprecipitated in cells expressing both receptors [22], demonstrating that CB_1_R forms receptor heteromers with DOR. CB_1_R activity is increased in cortical membranes from mice with a genetic deletion of DOR, and CB_1_R activity is reduced when cells endogenously expressing CB_1_R are transfected with DOR [22], suggesting that these receptors normally inhibit each other's activity. Moreover, CB_1_R signaling is reduced in the presence of low concentrations of DOR ligands in cells co-expressing both receptors [22]. Together, these data suggest that CB_1_R directly interacts with DOR, and that occupancy of one receptor may allosterically alter the activity of the other receptor within this receptor heteromer. As such, we examined whether CB_1_R and DOR interact in endogenous tissue. Furthermore, given that several studies have shown that opioid and cannabinoid receptor expression is dynamically regulated during neuropathic pain, we also examined the degree to which this interaction regulates receptor activity during a pathologic state, neuropathic pain.

Previous studies, under a variety of experimental conditions in rodents, have focused mostly on pain-associated changes in the expression of these receptors within primary afferents/dorsal root ganglia (DRG) or within spinal cord. Within one or two days after a peripheral nerve lesion, opioid receptor mRNA and protein, particularly for the mu opioid receptor (MOR) and DOR, are upregulated within DRG and within the injured nerve proximal to the lesion site, indicating increased transport of opioid receptors to the periphery [13], [28]–[33]. However, a decrease in receptor levels has been reported at later time points after the lesion [28], [34]–[36]. Within the dorsal horn of the spinal cord, MOR and DOR levels have been reported to increase transiently [37], [38] immediately after peripheral nerve lesion, but are unchanged or slightly decreased relative to control levels by 7 days and at time points up to 4 weeks following a lesion [34], [36]–[45]. Decreases in MOR and DOR levels in the spinal cord could be a consequence of receptor activation and internalization in response to enhanced release of endogenous opioid peptides. Unlike opioid receptors, CB_1_R levels in DRG and spinal cord are reportedly unchanged immediately after peripheral nerve lesion, but are consistently increased during chronic neuropathic pain [33], [46]–[49]. Interestingly, animals with increased CB_1_R expression show enhanced analgesic responses to a CB_1_R agonist, suggesting that the increase in receptor levels could have a protective function [46], [48], [50].

While most studies have focused on neuropathic pain-associated changes in receptor expression within peripheral nerves, DRGs or spinal cord, few have examined alterations within supraspinal regions, despite clear evidence that neural activity within thalamus, cerebral cortex, and amygdala, is altered during neuropathic pain [51], [52]. Among these brain regions, the cerebral cortex and its subregions play a prominent role in pain perception and response. Altered opioid and cannabinoid receptor activity in cerebral cortex may contribute to changes in neuronal signaling and mood states during neuropathic pain. Previous studies have shown that activation of these receptors can affect affective states. For example, administration of DOR agonists or enkephalinase inhibitors (the latter leading to increases in endogenous opioids) reduces depressive-like and anxiety-like behavior [16], [17], [53], [54], while systemic or cingulate cortex injection of DOR antagonists heightens anxiety [16], [55], [56]. In addition, genetic deletion of DOR increases levels of anxiety and depressive-like behavior in mice [57]. Similarly, systemic or prefrontal cortical (PFC) injection of CB_1_R agonists produces antidepressant effects and extinction of conditioned fear responses [18], [19], while low doses of CB_1_R agonists [58] or inhibitors of endocannabinoid degrading enzymes [59], [60] can induce anxiolytic effects. Based on this work, it is clear that cortical activation of DOR and CB_1_R can positively modulate affective states. Therefore, these receptors are suitable targets for alleviation of depression, anxiety and fear during neuropathic pain.

A few studies have examined changes in supraspinal opioid and cannabinoid receptor expression and activity during neuropathic pain. In the case of DOR, one study reported no change in DOR activity in lower midbrain and limbic forebrain [61] while another study found reduced DOR activity in the frontal cortex of nerve lesioned mice [55]. Stimulation of DOR is anxiolytic, so a reduction in DOR activity may underlie a heightened anxiety state. In the case of CB_1_R, no change in receptor binding was observed in anterior singulate cortex following peripheral nerve lesion, but CB_1_R activity was reduced 10 days after injury [62]. It has recently been demonstrated that, in the anterior singulate cortex, CB_1_R protein levels initially decrease 7 days after spinal cord injury, then increase by 42 days [63]. Therefore, a clear picture of the time course and degree to which cortical opioid and cannabinoid receptor expression and activity are changed during neuropathic pain has not yet emerged. To address this question, we examined changes in cortical DOR and CB_1_R expression, activity and interaction at several time points subsequent to a peripheral nerve lesion. We observed that, while cortical CB_1_R and DOR protein levels increase after peripheral nerve lesion, CB_1_R activity increases but DOR activity decreases. Because reduced cortical DOR activity during neuropathic pain may underlie heightened anxiety states, we questioned whether heteromerization between CB_1_R and DOR would diminish DOR activity, and whether this could be restored pharmacologically by CB_1_R ligands. We found that low, non-signaling doses of CB_1_R ligands (agonist or antagonist) could allosterically enhance DOR binding and activity, and, given that this effect is blocked by a heteromer specific antibody, that this enhancement is mediated by the CB_1_R-DOR heteromer. These results are consistent with allosteric modulation of DOR activity by CB_1_R within the CB_1_R-DOR heteromer, and suggest that cortical CB_1_R-DOR heteromer is a suitable target for blockade of neuropathic pain- associated negative mood states.

## Results

### Neuropathic Pain Model

**Figure 1:**
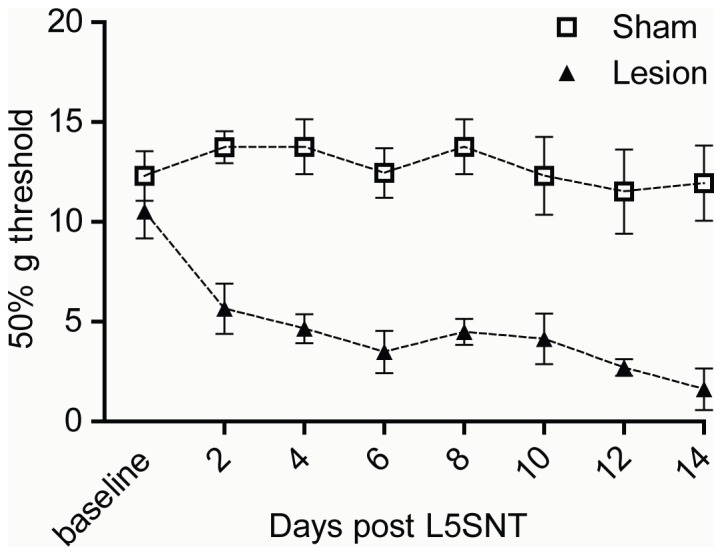
Lesioned animals experience mechanical allodynia. Mechanical response threshold to Von Frey fibers was measured before and after surgery in sham and L5SNT lesioned animals. Response threshold value was calculated as described in “Methods”. Data represent Mean ± SEM (n = 12 animals/group).

We used a common peripheral nerve lesion model, L5 spinal nerve ligation/transection (L5SNT) [64], [65], to produce neuropathic pain in adult male Sprague-Dawley rats. In this model, the right L5 spinal nerve is exposed, ligated and transected 1 mm distal to the ligation. For “sham” animals, the L5 spinal nerve is exposed, but is neither ligated nor transected. Using the Von Frey test [66], [67], we measured the presence of mechanical allodynia before and up to 14 days after surgery and found that lesioned animals show a lower threshold of response within two days after surgery (**Fig. 1**). This decrease in response threshold is maintained in lesioned animals throughout the 14 day testing period; sham animals do not demonstrate altered mechanical pain thresholds (**Fig. 1**). Therefore, L5SNT surgery produces neuropathic pain that is long-lasting.

### Altered receptor expression and activity in cortex of lesioned animals

**Figure 2:**
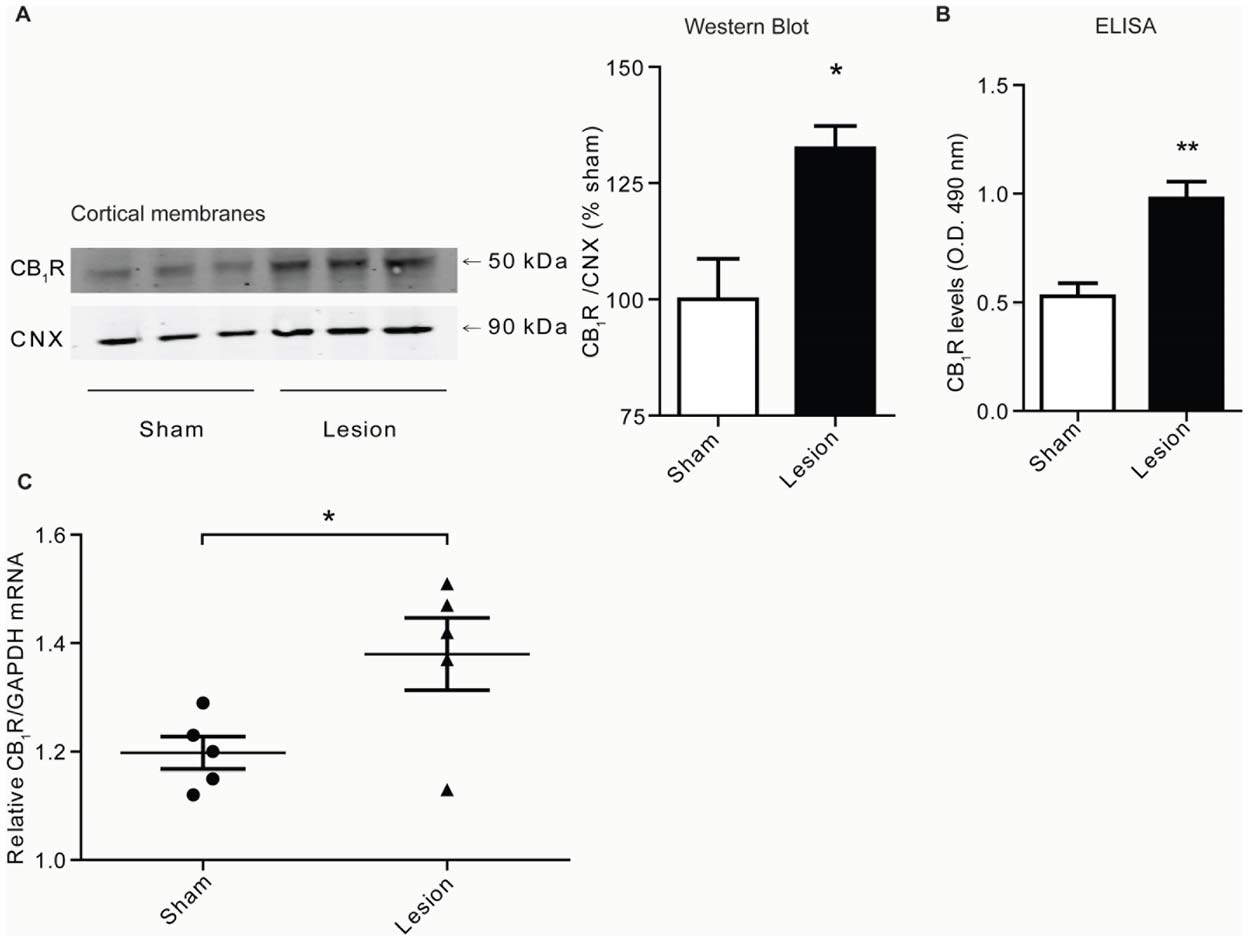
CB_1_R levels increase in cortex of lesioned animals. **A,** Representative Western Blot and quantification for CB_1_R and CNX (Calnexin) in cortical membranes from 3 individual sham or lesioned animals, 14 days after surgery. Data represent Mean ± SEM (n = 6 animals/group). **B,** ELISA data for CB_1_R detection using a CB_1_R specific antibody in cortical membranes from sham and lesioned animals 14 days after surgery. Data represent Mean ± SEM (n = 4 animals/group). Statistically significant differences between sham and lesion groups are indicated*, p<0.05; **, p<0.01 (t-test). **C,** RT-PCR for CB_1_R (measured relative to GAPDH) in cortical preparations from sham and lesioned animals 14 days after surgery. Data represent Mean ± SEM (n = 5 animals/group). Statistically significant differences between sham and lesion groups are indicated *, p<0.05 (t-test).

**Figure 3:**
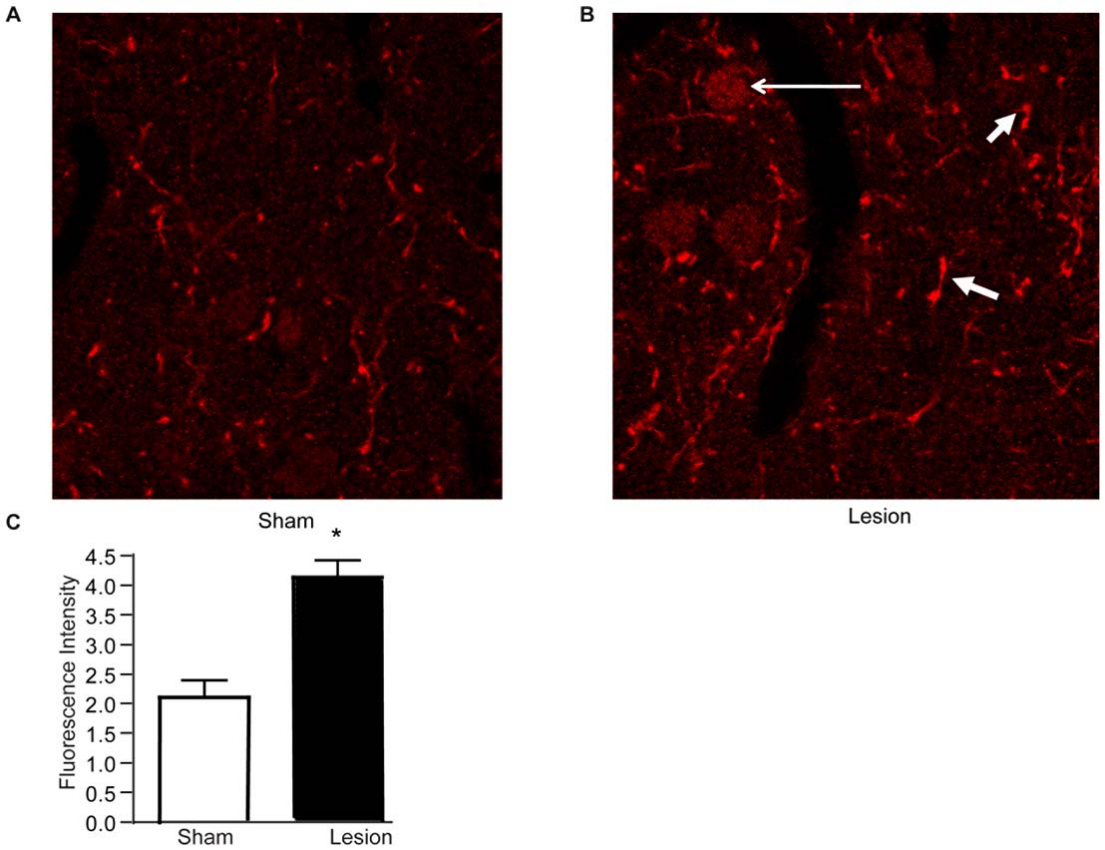
CB_1_R levels are increased in cortex of lesioned animals as visualized by immunohistochemistry. **A,B** A representative pair of images from studies examining the localization of CB_1_R by immunohistochemistry in layer II/III of PFC of sham and lesioned animals 14 days after surgery. Representative of 8 images taken per condition is shown. Scale bar = 20 µm. Increased CB_1_R immunoreactivity is indicated by short arrows and cell bodies are indicated by the long arrow. **C,** Quantification of changes in CB_1_R fluorescence intensity in PFC. Data represents mean fluorescence intensity from 16 images (4 per animal, n = 2 animals/group; p<0.05, t-test).

Next, changes in levels of CB_1_R and DOR in various brain regions of animals with peripheral nerve lesion were investigated. We found that, when compared to sham animals, CB_1_R levels are increased in cortical membranes prepared from brains of lesioned animals 14 days after surgery (**Fig. 2A–C**) as measured by Western blot, ELISA and RT-PCR. The increase in CB_1_R levels is only evident 14 days after surgery, and not at 3 or 7 days (**Table 1**), indicating that alterations in cortical CB_1_R receptor levels are not synchronous with the development of mechanical allodynia. We further found significant increases in CB_1_R levels in some, but not all, brain regions. For example, there was an increase in hypothalamus and midbrain, but not in striatum, hippocampus and cerebellum (**Table 2**). Taken together, these results indicate that CB_1_R is selectively upregulated in specific brain regions in response to nerve lesion. Next, we focused on the cortex and examined whether the relative distribution of CB_1_R was altered in response to nerve lesion. This was evaluated 14 days after nerve lesion by immunohistochemistry. In layer II/III of PFC, a region of interest as it normally expresses high levels of CB_1_R, a synaptic- and neuritic-like staining pattern is evident in sections from both sham and lesioned animals (**Fig. 3A–B**). Furthermore, the density of CB_1_R expression was found to be higher in the cortex of lesioned animals (**Fig. 3B–C**). Interestingly, lesioned animals show increased CB_1_R expression in cell bodies, which suggests that the receptor is redistributed and/or new receptors are synthesized during neuropathic pain.

**Table 1:** Time course of changes in CB_1_R and DOR levels in membranes from sham vs. lesioned animals.

Days post-surgery	CB_1_R	DOR
**3 days**		
**Sham**	100.0±21.32	100.0±46.67
**Lesion**	102.4±17.84	50.37±18.50
**Statistics**	*p* = 0.9369	*p* = 0.3789
**7 days**		
**Sham**	100.0±12.73	100.0±13.02
**Lesion**	102.1±20.68	100.1±40.77
**Statistics**	*p* = 0.9348	*p* = 0.9982
**14 days**		
**Sham**	100.0±8.766	100.0±21.03
**Lesion**	132.5±4.801	280.9±24.50
**Statistics**	*p* = 0.0314 *(* * *)*	*p* = 0.0050 *(* ** *)*

Levels of CB_1_R or DOR were measured by Western blot analysis as described in “Methods”. p-values from t-tests comparing mean CB_1_R or DOR levels (percent relative to CNX and normalized to sham) in cortical membranes of sham vs. lesioned animals (n = 6 animals/group) at different time points after surgery. Statistically significant differences between sham and lesion groups are indicated.

* , p<0.05,

** p<0.01 (t-test).

**Table 2:** Changes in CB_1_R, DOR and CB_1_R-DOR levels in membranes from sham vs. lesioned animals.

Region	CB_1_R	DOR	CB_1_R-DOR
**Striatum**			
**Sham**	100.0±9.18	100.0±4.74	100.0±12.27
**Lesion**	143.8±14.38	147.0±11.02	127.4±17.78
**Statistics**	*p* = 0.0572	*p* = 0.0173 *(* * *)*	*p* = 0.2737
**Hippocampus**			
**Sham**	100.0±2.59	100±1.98	100±9.72
**Lesion**	85.51±8.05	96.76±8.46	88.26±6.08
**Statistics**	*p* = 0.1367	*p* = 0.7240	*p* = 0.3636
**Cerebellum**			
**Sham**	100.0±26.41	100.0±23.41	100.0±19.59
**Lesion**	116.2±21.58	114.3±31.39	155.4±37.20
**Statistics**	*p* = 0.6592	*p* = 0.7346	*p* = 0.2580
**Hypothalamus**			
**Sham**	100.0±4.34	100.0±4.96	100.0±5.76
**Lesion**	127.3±4.59	128.4±8.72	155.5±5.14
**Statistics**	*p* = 0.0124 *(* * *)*	*p* = 0.0471 *(* * *)*	*p* = 0.0020 *(* *** *)*
**Midbrain**			
**Sham**	100.0±11.65	100.0±16.65	100.0±8.20
**Lesion**	169.4±14.39	131.1±1.70	156.0±4.09
**Statistics**	*p* = 0.0200 *(* *** *)*	*p* = 0.1368	*p* = 0.0036 *(* ** *)*

Changes in CB_1_R, DOR or CB_1_R-DOR levels were detected in different brain regions by ELISA as described in “Methods”. p-values from t-tests comparing mean CB_1_R, DOR or CB_1_R-DOR levels (normalized to sham, percent of sham listed) in membranes from various brain regions of sham vs. lesioned animals (n = 6 animals/group) 14 days after surgery are shown. Statistically significant differences between sham and lesion groups are indicated.

* , p<0.05,

** , p<0.01,

*** , p<0.001 (t-test).

**Figure 4:**
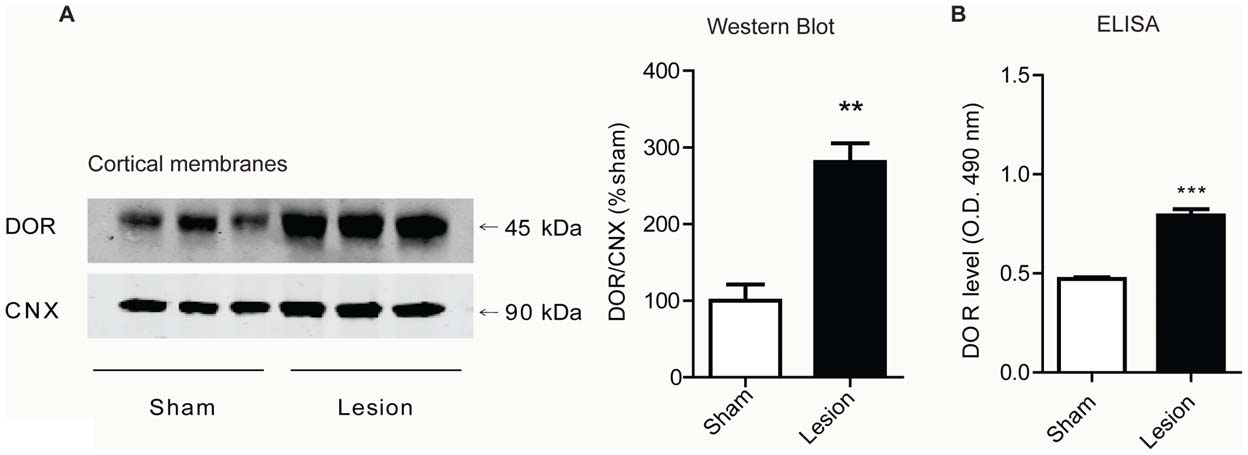
DOR levels increase in cortex of lesioned animals. **A,** Representative Western Blot and quantification for DOR and CNX in cortical membranes from 3 individual sham or lesioned animals. Data represent Mean ± SEM (n = 6 animals/group). Statistically significant differences between sham and lesion groups are indicated **, p<0.01 (t-test). **B,** ELISA data for DOR in cortical membranes from sham and lesioned animals. Data represent Mean ± SEM (n = 4 animals/group). Statistically significant differences between sham and lesion groups are indicated ***, p<0.001 (t-test).

Next, we examined if the L5SNT nerve lesion led to changes in DOR levels. As in the case of CB_1_R, we find increases in DOR levels in cortical membranes prepared from the brains of lesioned animals, but not sham animals, 14 days after surgery (**Fig. 4A–B**). This increase is only evident 14 days after surgery, not at 3 or 7 days (**Table 1**), similar to what was found for CB_1_R. DOR levels are also differentially regulated in other brain regions 14 days after surgery; we observed significant increases in hypothalamus and in striatum, but not in hippocampus, cerebellum and midbrain (**Table 2**). Taken together, these results indicate that DOR levels are also selectively increased in specific brain regions in response to nerve lesion.

**Figure 5:**
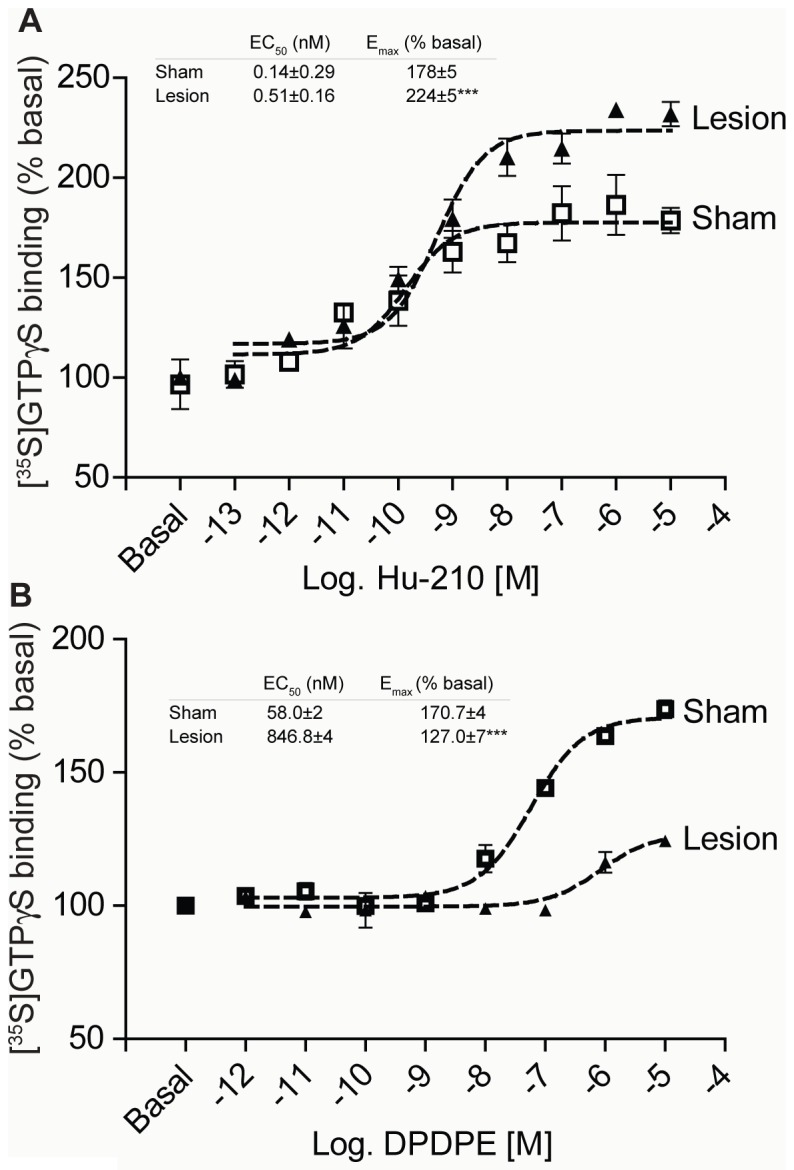
CB_1_R activity increases while DOR activity decreases in cortex of lesioned animals. **A,** [^35^S]GTPγS binding was carried out with cortical membranes from sham and lesioned animals. Membranes from cortices were prepared as described in “Methods” and treated with 0.1 pM – 10 µM Hu-210 for 1 hour. [^35^S]GTPγS binding to membranes was detected using a scintillation counter. Basal [^35^S]GTPγS binding in vehicle treated membranes is taken as 100%. Data represent Mean ± SEM (n = 3 individual animals in triplicate). **B,** Membranes from cortices of sham and lesioned animals were treated with 1 pM – 10 µM DPDPE for 1.5 hours. [^35^S]GTPγS binding to membranes was detected using a scintillation counter. Basal [^35^S]GTPγS binding in vehicle treated membranes is taken as 100%. Data represent Mean ± SEM (n = 3 individual animals in triplicate).

Next, we examined if increased receptor levels were accompanied by increases in receptor activity using the [^35^S]GTPγS assay, which measures coupling between the receptor and G-protein. We find that CB_1_R can be activated by a specific ligand, Hu-210, in cortical membranes from sham and lesioned animals, and that maximal activity is higher in cortical membranes from lesioned animals (∼178% for sham and ∼224% for lesioned) (**Fig. 5A**). Thus, 14 days after surgery, lesioned animals demonstrate increased CB_1_R mRNA, protein levels and receptor activity in cortex.

Changes in DOR activity using [D-Pen^2,5^]Enkephalin , [D-Pen^2^,D-Pen^5^]Enkephalin (DPDPE) as a receptor specific ligand were also examined. Unexpectedly, we found a decrease in maximal DOR activity in cortical membranes from lesioned animals when compared with sham animals (∼171% for sham and ∼127% for lesioned) (**Fig. 5B**). This result was somewhat surprising, as DOR activity was expected to increase in the cortex of lesioned animals commensurate with the documented increases in receptor protein. One possible explanation for the decrease in DOR activity despite an increase in DOR protein is that DOR activity is suppressed by altered interactions with other proteins. This is consistent with previous reports that demonstrated direct, antagonistic interactions between CB_1_R and DOR *in vitro* [22], [27].

### Neuropathic pain-associated alterations in CB_1_R-DOR levels

**Figure 6:**
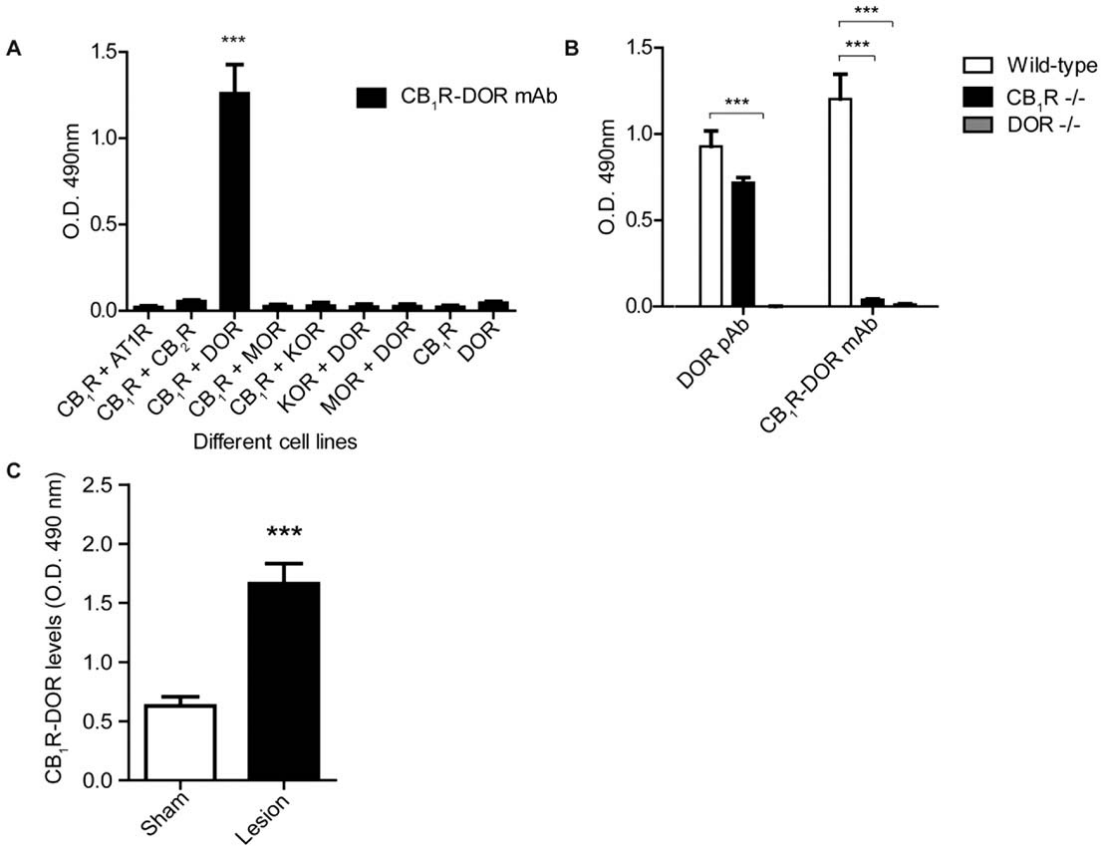
CB_1_R-DOR antibody specificity and increases in CB_1_R-DOR levels during neuropathic pain. **A,** ELISA for mouse monoclonal CB_1_R-DOR antibody using the following cell lines: 1) HEK cells expressing the following receptor combinations: DOR, KOR+DOR, or MOR+DOR; 2) N2A cells (which endogenously express CB_1_R) alone or co-expressing the following receptors: AT1R, CB2R, DOR, MOR, or KOR. Data represent Mean ± SEM (n = 3 independent experiments in triplicate). Statistically significant differences between sham and lesion groups are indicated ***, p<0.001 (t-test). **B,** ELISA for rat polyclonal DOR, and mouse monoclonal CB_1_R-DOR antibodies in cortical membranes from wild type, CB_1_R −/−, and DOR −/− animals. Data represent Mean ± SEM (n = 3 animals/group). Statistically significant differences between wild type and −/− groups are indicated ***, p<0.001 (t-test). **C,** ELISA for CB_1_R-DOR in cortical membranes from sham and lesioned animals. Data represent Mean ± SEM (n = 4 animals/group). Statistically significant differences between sham and lesion groups are indicated ***, p<0.001 (t-test).

Our data suggest that the abundance of CB_1_R-DOR heteromers may increase after nerve ligation surgery, given that the levels of both receptors individually increase in the cortex of lesioned animals. In order to directly probe the presence of and changes in the levels of CB_1_R-DOR heteromers, a specific mouse monoclonal antibody directed against the CB_1_R-DOR heteromer was generated using a previously employed subtractive immunization strategy [25], [26]. Among twelve clones that exhibited specificity towards CB_1_R-DOR we selected one for further characterization. Hereafter this antibody will be referred to simply as CB_1_R-DOR mAb. (**Fig. 6**). The CB_1_R-DOR mAb only detects an epitope in cells expressing both CB_1_R and DOR, but not in cells expressing the individual receptors (**Fig. 6A**). More importantly, the antibody does not recognize an epitope when CB_1_R is co-expressed with receptors other than DOR and vice versa (**Fig. 6A**). Finally, the CB_1_R-DOR mAb only detects an epitope in cortical membranes from wild type, but not CB_1_R −/− or DOR −/− mice (**Fig. 6B**). Next we examined CB_1_R-DOR levels in various brain regions 14 days after surgery and found that they are upregulated in some, but not all, brain regions: significant increases were observed in cortex (**Fig. 6C**), hypothalamus and midbrain, and no changes in striatum, hippocampus and cerebellum (**not shown**).

### Allosteric modulation of DOR by interaction with CB_1_R during neuropathic pain

**Figure 7:**
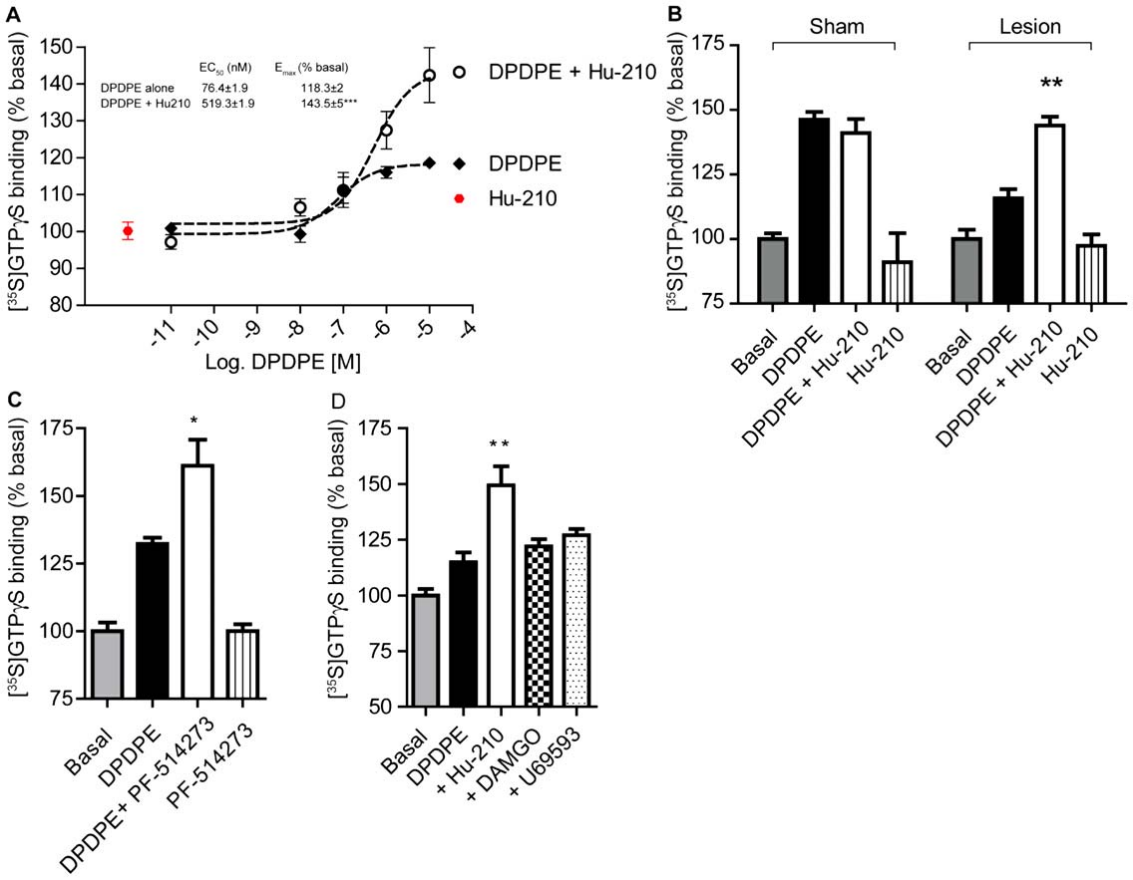
DOR activity is enhanced in the presence of CB_1_R ligands in cortical membranes from lesioned animals. **A,** Membranes from cortices of lesioned animals were treated with 10 pM – 10 µM DPDPE in the absence of presence of 1 pM Hu-210, or with 1 pM Hu-210 alone for 1.5 hours. [^35^S]GTPγS binding to membranes was detected using a scintillation counter. Basal [^35^S]GTPγS binding in vehicle treated membranes is taken as 100%. Data represent Mean ± SEM (n = 3 individual animals in triplicate). Statistically significant differences between 10 µM DPDPE alone and 10 µM DPDPE+1 pM Hu-210 are indicated ***, p<0.001, (t test). **B,** Membranes from cortices of sham and lesioned animals were treated with 10 µM DPDPE in the absence of presence of 1 pM Hu-210, or with 1 pM Hu-210 alone for 1.5 hours. [^35^S]GTPγS binding to membranes was detected using a scintillation counter. Basal [^35^S]GTPγS binding in vehicle treated membranes is taken as 100%. Data represent Mean ± SEM (n = 4 individual animals in triplicate). Statistically significant differences between 10 µM DPDPE alone and 10 µM DPDPE+1 pM Hu-210 are indicated **, p<0.01, (t test). **C,** Membranes from cortices of lesioned animals were treated with 10 µM DPDPE in the absence of presence of 1 µM PF-514273, or with 1 µM PF-514273 alone for 1.5 hours. [^35^S]GTPγS binding to membranes was detected using a scintillation counter. Basal [^35^S]GTPγS binding in vehicle treated membranes is taken as 100%. Data represent Mean ± SEM (n = 3 individual animals in triplicate). Statistically significant differences between 10 µM DPDPE alone and 10 µM DPDPE+1 µM PF-514273 are indicated *, p<0.05, (t test). **D,** Membranes from cortices of lesioned animals were treated with 10 µM DPDPE in the absence of presence of 1 pM Hu-210, or 10 nM DAMGO or 10 nM U69593 for 1.5 hours. [^35^S]GTPγS binding to membranes was detected using a scintillation counter. Basal [^35^S]GTPγS binding in vehicle treated membranes is taken as 100%. Data represent Mean ± SEM (n = 3 individual animals in triplicate). Statistically significant differences between 10 µM DPDPE and 10 µM DPDPE+ligand are indicated **, p<0.01, (t test).

**Figure 8:**
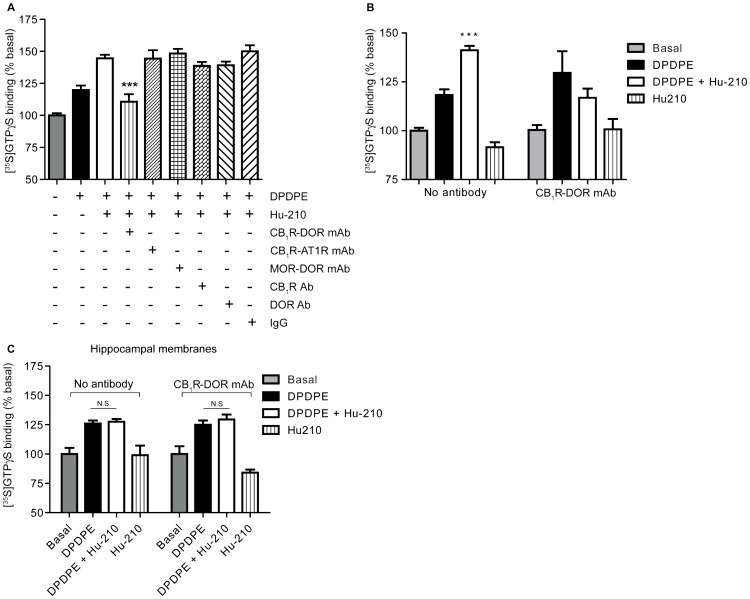
CB_1_R-DOR heteromer-specific antibody blocks enhancement of DOR activity. **A,** Membranes from cortices of lesioned animals were treated with 10 µM DPDPE without or with Hu-210 in the absence or presence of 1 µg of indicated antibodies. [^35^S]GTPγS binding to membranes was detected using a scintillation counter. Basal [^35^S]GTPγS binding in vehicle treated membranes is taken as 100%. Data represent Mean ± SEM (n = 3 individual animals in triplicate). Statistically significant differences between 10 µM DPDPE+1 pM Hu-210 and 10 µM DPDPE+1 pM Hu-210+1 µg antibody are indicated ***, p<0.001, (t test). **B,** In the absence or presence of 1 µg CB_1_R-DOR heteromer-specific antibody, membranes from cortices of lesioned animals were treated with 10 µM DPDPE without or with 1 pM Hu-210. [^35^S]GTPγS binding to membranes was detected using a scintillation counter. Basal [^35^S]GTPγS binding in vehicle treated membranes is taken as 100%. Data represent Mean ± SEM (n = 3 individual animals in triplicate). Statistically significant differences between 10 µM DPDPE and 10 µM DPDPE+1 pM Hu-210 are indicated ***, p<0.001, (t test). **C,** Membranes from hippocampi of lesioned animals were treated with 10 µM DPDPE in the absence of presence of 1 pM Hu-210 for 1.5 hours. Membranes were also treated with 10 µM DPDPE and 1 pM Hu-210 in the presence of 1 µg of CB_1_R-DOR mAb. [^35^S]GTPγS binding to membranes was detected using a scintillation counter. Basal [^35^S]GTPγS binding in vehicle treated membranes is taken as 100%. Data represent Mean ± SEM (n = 3 individual animals in triplicate). n.s. not significant.

Allosteric modulation of CB_1_R on DOR activity was specifically explored by examining if a CB_1_R agonist could restore the suppressed DOR activity in cortical membranes from lesioned animals. For this, low, non-signaling doses of a CB_1_R agonist were used to examine whether occupancy, but not activation, of CB_1_R is sufficient to alter DOR activity. In the presence of Hu-210, a CB_1_R agonist, DOR activity in cortical membranes from lesioned animals increased (**Fig. 7A & B**); however Hu-210 had no effect on DOR activity in cortical membranes from sham animals (**Fig. 7B**). Whether occupancy of CB_1_R by an antagonist is sufficient to restore DOR activity was also examined. We found that the CB_1_R antagonist PF-514273 also enhanced DPDPE-simulated DOR activity in the cortex of lesioned animals (**Fig. 7C**). Neither MOR, nor kappa opioid receptor (KOR) specific ligands altered cortical DOR activity (**Fig. 7D**). Furthermore, treatment with a low, non-signaling dose of Hu-210 did not enhance DOR activity in membranes from hippocampus (**Fig. 8** **; see panel c**), a brain region in which CB_1_R and DOR levels do not change during neuropathic pain (**Table 2**). These results are consistent with the idea that the decrease in DOR activity in the cortex of neuropathic animals is due to an interaction with CB_1_R, and that this antagonistic interaction can be reversed by occupancy of CB_1_R. Together, the data suggests that occupancy of CB_1_R (by an antagonist or a low, non-signaling doses of selective agonist) in cortex of lesioned animals is sufficient to allosterically modulate DOR activity.

### CB_1_R-DOR heteromer-specific antibody blocks enhancement of DOR activity by CB_1_R ligands

In order to directly test whether the allosteric modulation of DOR activity by CB_1_R ligands is specific to the receptors within the CB_1_R-DOR heteromer, the CB_1_R-DOR heteromer selective antibody was used. The CB_1_R-DOR mAb selectively blocked Hu-210-mediated increases in DOR activity (**Fig. 8A**). In contrast, antibodies directed against other heteromer pairs (including MOR-DOR mAb [25] or CB_1_R-AT1R mAb [26]) or antibodies to CB_1_R or DOR alone did not block Hu-210 induced increases in DOR activity (**Fig. 8A**). The CB_1_R-DOR mAb did not alter basal [^35^S]GTPγS binding, nor did it alter DPDPE-stimulated DOR activity (**Fig. 8B**). Finally, CB_1_R-DOR mAb had no effect on DOR activity in the absence or presence of Hu-210 in membranes from hippocampus, a region in which neither CB_1_R, DOR, nor CB_1_R-DOR expression changed 14 days after lesion (**Fig. 8C**; **Table 2**). Together, these results support the idea that the potentiation of DOR activity by a low dose of Hu-210 is specific to regions in which CB_1_R-DOR heteromer expression is enhanced during neuropathic pain; the CB_1_R-DOR mAb only blocked heteromer-mediated signaling in those regions that showed an increase in CB_1_R-DOR expression.

### DOR binding is enhanced by interaction with CB_1_R within CB_1_R-DOR heteromer

**Figure 9:**
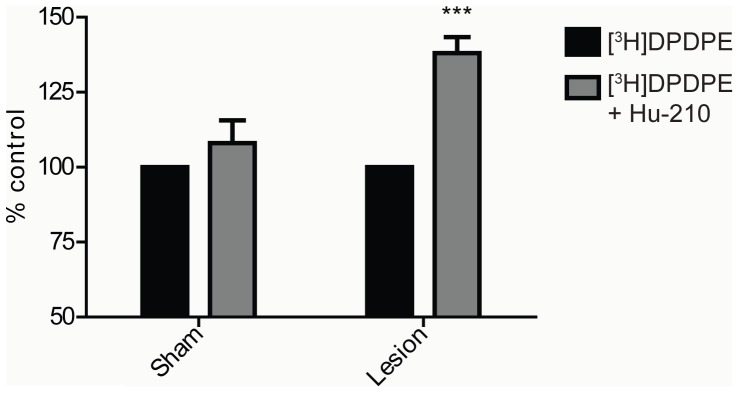
DOR binding is enhanced in the presence of low dose CB_1_R ligand in cortical membranes. Membranes from cortices of sham and lesioned animals were treated with 0.5 nM [^3^H]DPDPE in the presence or absence of 1 pM Hu-210 for 1 hour. Ligand binding assay was carried out as described in “Methods” and [^3^H]DPDPE binding to membranes was detected using a scintillation counter. Data represent Mean ± SEM (n = 7 animals/group). Statistically significant differences between [^3^H]DPDPE alone and [^3^H]DPDPE+1 pM Hu-210 are indicated ***, p<0.001, (t test).

Next, the allosteric modulation of DOR activity by occupancy of CB_1_R using ligand binding assays was examined. In the cortical membranes from sham animals, a low dose of Hu-210 did not alter [^3^H]DPDPE binding to DOR (**Fig. 9**). However, in cortical membranes from lesioned animals, the same dose of Hu-210 significantly enhanced [^3^H]DPDPE binding to DOR (**Fig. 9**). These results show that the occupancy of CB_1_R leads to an allosteric modulation of DOR conformation that allows increased DOR binding by its selective ligand.

**Figure 10:**
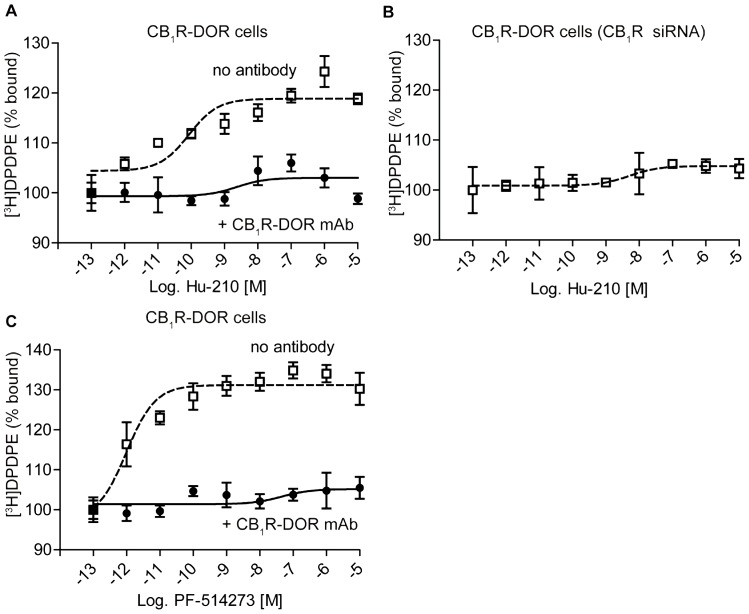
DOR binding is enhanced by CB_1_R ligands in membranes of cells expressing CB_1_R and DOR. **A,** Membranes from N2A-DOR cells were incubated with 100 fM – 10 µM Hu-210 in the presence of 0.5 nM [^3^H]DPDPE ± 1 µg CB_1_R-DOR monoclonal antibody for 1 hour. Ligand binding assay was carried out as described in “Methods” and [^3^H]DPDPE binding to membranes was detected using a scintillation counter. Data represent Mean ± SEM (n = 3 experiments in triplicate). **B,** Membranes from N2A-DOR cells in which CB_1_R expression was decreased by siRNA transfection were treated with 100 fM – 10 µM Hu-210 in the presence of 0.5 nM [^3^H]DPDPE. Ligand binding assay was carried out as described in “Methods” and [^3^H]DPDPE binding to membranes was detected using a scintillation counter. Data represent Mean ± SEM (n = 3 experiments in triplicate). **C,** Membranes from N2A-DOR cells were treated with 100 fM – 10 µM PF-514273 in the presence of 0.5 nM [^3^H]DPDPE along with the presence or absence of 1 µg CB_1_R-DOR monoclonal antibody for 1 hour. Ligand binding assay was carried out as described in “Methods” and [^3^H]DPDPE binding to membranes was detected using a scintillation counter. Data represent Mean ± SEM (n = 3 experiments in triplicate).

Whether DOR binding was allosterically modulated by CB_1_R was also examined using a cell culture model that allowed for manipulation of CB_1_R expression. In N2A-DOR cells, which endogenously express CB_1_R and are stably transfected with DOR, the binding of DOR ligand was enhanced in the presence of Hu-210 in a dose dependent fashion (**Fig. 10A**). This enhancement was blocked by the CB_1_R-DOR mAb (**Fig. 10A**) or by knocking down CB_1_R expression (**Fig. 10B**). This indicates that enhancement of DOR binding by Hu-210 requires the presence of CB_1_R and is mediated through the CB_1_R-DOR heteromer. We also examined if CB_1_R occupancy is sufficient to alter DOR binding, and found that treatment with the CB_1_R antagonist PF-514273 also enhanced [^3^H]DPDPE binding in a dose dependent fashion, and was blocked by addition of 1 µg of CB_1_R-DOR mAb (**Fig. 10C**). Together, these results indicate that enhancement of DOR activity requires the presence of CB_1_R, occupancy of CB_1_R, and is mediated through the CB_1_R-DOR heteromer.

## Discussion

The present study reveals that neuropathic pain-induced suppression of DOR activity can be reversed through allosteric modulation of the CB_1_R-DOR heteromer. Specifically, we showed that 1) DOR and CB_1_R expression increased in select brain regions 14 days after peripheral nerve lesion; 2) DOR activity decreased, while CB_1_R activity increased in the cortex of lesioned animals, suggesting that CB_1_R activity may suppress DOR activity; 3) Treatment with a CB_1_R antagonist or a low, non-signaling dose of a CB_1_R agonist could restore suppressed cortical DOR activity back to normal levels, an indication that, under certain conditions, CB_1_R can allosterically modulate DOR activity and binding; and 4) Modulation of DOR activity by CB_1_R occurs within the CB_1_R-DOR heteromer, as a heteromer-specific antibody was able to block allosteric enhancement of DOR activity. These results demonstrate an important role for CB_1_R-DOR heteromer formation in cortex during neuropathic pain. Though further studies will be necessary, the present findings suggest that a heteromer-directed therapeutic strategy for enhancing DOR activity could potentially be utilized to reduce neuropathic pain and associated anxiety behaviors.

The L5SNT peripheral nerve lesion model leads to the production of neuropathic pain, and our results show that mechanical allodynia in lesioned animals develops quickly – within 2 days – and is persistent, lasting at least 14 days. Previous studies have shown the development of mechanical allodynia [47], [66]–[68], thermal hyperalgesia [45], central sensitization [69], and spontaneous C-fiber activity [70] using this pain model. Interestingly, a recent study found that spinal nerve ligated rodents do not develop anxiety or depression related behaviors until 15 days after ligation, although sensory hyperalgesia develops much earlier [71]. Our finding, that changes in cortical DOR and CB_1_R expression are not observed at 3 or 7 days after surgery but only at 14 days, highlight the possibility that changes in cortical receptor expression and activity underlie the development of behavioral phenomena such as anxiety and depression.

We also observed increases in DOR and CB_1_R levels using cortical membrane preparations. While this is the first study to examine changes in cortical levels of these receptors during neuropathic pain, previous human studies found increases in CB_1_R levels in prefrontal cortex during depression or after suicide [72], [73], suggesting that CB_1_R upregulation in this region could underlie negative mood states. It is unclear whether increased receptor levels reflect increased receptor synthesis or redistribution of receptors to a particular membrane compartment. Previous studies have shown that pain-induced release of inflammatory mediators causes increased plasma membrane insertion of DOR in DRGs and spinal cord [11], [74], though these changes occurred early (after 3 days) and likely underlie the effectiveness of DOR agonists as analgesic, not as anxiolytic or antidepressant agents. The long term nature of DOR and CB_1_R upregulation in our studies (both DOR and CB_1_R levels are increased after 14 days, but not after 3 or 7 days), is suggestive of increased receptor synthesis. Moreover, our finding that lesioned animals showed increases in receptor staining in cortical cell bodies and processes suggests that new receptors are being synthesized and trafficked to neuronal processes. Previous studies have examined CB_1_R expression throughout the brain and found that the receptor is mostly localized to axons and synaptic terminals (reviewed in [75]). Our finding, that CB_1_R is localized in cell bodies, in addition to neuritic-like processes and synaptic-like puncta, suggests a new pattern of distribution for the receptor during a disease state. It is also possible that changes in DOR expression might be driving changes in CB_1_R localization (and vice versa). Studies in heterologous cells demonstrated that a lack of (or a decrease in) DOR expression leads to intracellular trafficking of CB_1_R. Conversely, increasing the level of DOR results in localization of CB_1_R to the plasma membrane [22]. A similar mechanism might underlie the distribution and localization of CB_1_R in the cortex of animals with peripheral nerve lesion.

In addition to changes in receptor expression and localization in cortex during neuropathic pain, we found that CB_1_R, DOR and CB_1_R-DOR were upregulated in hypothalamus and midbrain during neuropathic pain. Stimulation of either CB_1_R or DOR in the midbrain, particularly in the PAG, can activate fibers that descend into the dorsal horn of the spinal cord and inhibit pain signals. Therefore, upregulation of these receptors in this brain region may be protective and may promote analgesia during neuropathic pain, although no studies have examined pain-associated changes in receptor activity in this region. The exact role of cannabinoid and opioid receptor expression in the hypothalamus is unclear, although activation of receptors in this region may promote feeding behavior and analgesia [75], [76].

In this study we provide evidence supporting the idea that CB_1_R directly interacts with DOR in cortex during neuropathic pain. For this, we developed a CB_1_R-DOR heteromer specific monoclonal antibody using the subtractive immunization strategy and show that the antibody is both highly selective and is able to quantify heteromer levels in the cortices of animals experiencing neuropathic pain. Hence the heteromer-selective antibody serves as a unique and powerful tool to demonstrate heteromer-specific activity.

Allosteric modulation of receptor activity by dimerization has increasingly been recognized [77]. In this study, we found that the occupancy of CB_1_R is sufficient to increase DOR ligand binding to its receptor. This indicates that the ligand occupancy-induced changes in CB_1_R conformation alters the interaction between CB_1_R and DOR such that DOR is better able to bind its own ligand. Previous studies have demonstrated that cannabidiol can reduce the rate of dissociation of [^3^H]naltrindole (a DOR selective ligand) from DOR [78], suggesting that high doses of cannabidiol or low doses of Hu-210 stabilize similar CB_1_R conformations. Finally, recent studies reported that the DOR ligands allosterically modulate MOR agonist binding and signaling, and this effect could be blocked by the application of a MOR-DOR-selective antibody [25], [79]. Together, these studies emphasize the allosteric nature of the interaction between receptor protomers that could be harnessed for the development of combination therapies [80], [81]. Based on our results, several pharmacological strategies for targeting CB_1_R-DOR during neuropathic pain could be proposed: 1) A combination of low doses of CB_1_R ligands with DOR agonists to increase the potency of selective DOR agonists in reducing anxiety during neuropathic pain; 2) A heteromer specific compound (e.g. bivalent ligand) composed of a weak CB_1_R ligand and full DOR agonist as a potent anxiolytic for neuropathic pain; and 3) Biologicals that block heteromer activity (such as, CB_1_R-DOR heteromer specific antibody or a peptide that selectively blocks CB_1_R-DOR interaction) to reduce neuropathic pain or possibly symptoms of anxiety and depression that associated with neuropathic pain. Strategy 1 (a low dose of DOR antagonists to enhance morphine effects) was employed successfully to enhance morphine analgesia in animal models [82], [83], [84]. Additionally, using animal models, strategy 2 (bivalent ligands composed of a DOR antagonist and MOR agonist) was shown to result in more potent analgesics that induced less tolerance than morphine [85]. Strategy 3 (biologicals that block heteromer activity ) has been used to disrupt MOR-DOR heteromers [86] or D1-D2 heteromers [87] or to block DOR-KOR heteromers [88]. We hypothesize that cortical injection of a peptide disrupting CB_1_R-DOR interactions or a drug specifically blocking this heteromer would prevent the decrease in DOR activity and concomitantly reduce the development of long-term anxiety in neuropathic animals. However, additional studies will be needed in order to fully test this hypothesis.

In summary, we have identified CB_1_R-DOR as a novel entity regulating receptor activity during a disease state. Furthermore, we have identified allosteric modulation of one receptor's activity by the other as a molecular mechanism to enhance the activity of the receptor compromised by the pathology. Such a mechanism can be targeted to develop therapeutics for enhancing the activity of disease-specific heteromers, particularly for those receptors whose activation is physiologically protective, but whose activity is suppressed by its partner receptor.

## Methods

### Ethics Statement

Animal studies were carried out according to protocols approved by the Mount Sinai School of

Medicine Institutional Animal Care and Use Committee (Permit # 02-0805).

### Animals

#### Rats

Adult male Sprague-Dawley rats (200–250 g) were used for all experimental groups (sham and lesion). Animals were maintained on a 12-hour light/dark cycle and were allowed to acclimatize to their environment one week prior to experimentation.

### Surgery

Rats subjected to L5 spinal nerve transection (L5SNT) were assessed on the VonFrey test on the day of surgery (prior to surgery) to establish a baseline response profile (see below for VonFrey protocol). Thereafter, animals were tested behaviorally every two days, and sacrificed 3, 7 or 14 days following L5SNT or sham surgery. Ten minutes prior to surgery, animals received an IM injection of ketamine (60 mg/kg)/xylazine (7.5 mg/kg) (Sigma-Aldrich, St. Louis, MO, USA). The surgical area was shaved and then cleaned with ethanol. The right paraspinal muscles (at lumbar levels L4–L6) were separated, and the L6 transverse vertebral process was exposed and partially removed. The L4 and L5 spinal nerves, which run below the L6 transverse process, were separated. The L5 spinal nerve was tightly ligated with a 5-0 silk suture and then transected 1 mm distal to the ligation (prior to entry into the sciatic nerve). Paraspinal muscles and superficial connective tissue were sutured (3-0 nylon) into their anatomical layers; skin at the incision site was sutured (3-0 nylon). Topical antibiotic (bacitracin, neomycin, and polymyxin B) was applied to the incision site. Animals were monitored until awakening from the anesthetic and then returned to their home cages. For sham animals, the L5 spinal nerve was exposed but was not ligated or transected. All other surgical steps were identical between animals in these two groups. Experimental group size was 5–6 animals.

### Von Frey Test

For mechanical allodynia, all animals (lesioned and sham) were placed onto a testing platform containing a metal, perforated floor (Stoelting Co., Wood Dale, IL, USA). After 15 min of acclimatization to the testing chamber, mechanical allodynia was assessed on both hindpaws using calibrated filaments (Stoelting Co., Wood Dale, IL, ISA). All trials began with the 2.04 g filament, and proceeded using up-down trial design and standard quantitative methods [89], [90]. Each filament was applied to the hindpaw six times per filament size. A positive response, defined as hind limb withdrawal or flinching on three of six tests or on two consecutive tests, to one filament was followed by the use of a smaller filament. Conversely, a negative response was followed by the use of larger filament. Each animal was tested with six filaments using the up-down method to determine the threshold of the mechanical response in the hindpaw.

### Membrane Preparation

Membranes were prepared from cortices of control, sham and L5SNT lesioned animals. Animals were sacrificed with CO_2_ gas at 3, 7 or 14 days after surgery and then rapidly decapitated. Brains were removed and the following anatomical subregions were dissected out: hypothalamus, prefrontal cortex, rest of neocortex, striatum, hippocampus, PAG, midbrain, rostral ventral medulla and cerebellum. To prepare membranes, dissected tissue was manually homogenized in cold sucrose buffer containing 50 mM Tris (pH 7.4), 100 mM NaCl, 0.2 mM EDTA, 3 mM MgCl_2_, 1 mM DTT and 250 mM sucrose. The homogenate was centrifuged at 1000 g for 5 min, the pellet was discarded and the supernatant was then centrifuged twice at 40,000 g for 10 min. The resulting pellet was resuspended in homogenizing buffer containing 50 mM Tris-Cl (pH 7.4), 100 mM NaCl, 3 mM MgCl_2_ and 0.2 mM EGTA. Membranes were stored at −80C. Membranes from DOR −/− mice [57] or CB_1_R −/− mice [92] were used as controls in ELISA experiments for validation of CB_1_R-DOR antibody.

### Western Blotting

40 µg of proteins from membranes (prepared as described above) were added to 6× Laemmli sample buffer. Proteins were resolved by 10% SDS-PAGE and were subjected to Western blot analysis using a rabbit polyclonal antibody against the C-terminus of CB_1_R (Cayman Chemical, Ann Arbor, MI, USA) (1∶300 dilution), a rat polyclonal antibody against DOR [22], [93] (1∶1000 dilution), or a rabbit polyclonal antibody against Calnexin (Cell Signaling, Boston, MA, USA) (1∶10,000 dilution), a resident ER membrane protein. Blotting and imaging with the Odyssey imaging system (LI-COR, Lincoln, NE, USA) was performed as per the manufacturer's protocols. The following secondary antibodies were utilized for visualization of the primary antibodies: goat-anti-rabbit IRDye 680 or goat-anti-rat IRDye 800 (both at a working dilution of 1∶10,000) (Rockland Immunochemicals, Gilbertsville, PA, USA). Band intensity was densitized using the Odyssey imaging system (LI-COR, Lincoln, NE, USA) software.

### CB_1_R-DOR antibody generation

The CB_1_R-DOR antibody was generated using a subtractive immunization strategy, essentially as described for the generation of our CB_1_R-AT1R antibody [26]. Mice were made tolerant to immunogenic epitopes in N2A cell membranes endogenously expressing CB_1_R by intraperitoneal injection. Tail bleeds were monitored for titers of antibody being produced. Upon exhibiting a decrease in antibody titer ,as measured by ELISA, mice were then given intraperitoneal injections of membranes from N2A cells expressing DOR, along with booster injections over the next 15 days. Spleen cells from animals secreting antibody were then fused with SP-20 myeloma cells to generate monoclonal antibodies. Clones secreting monoclonal antibodies were screened by ELISA as described [82] against the following cell lines: untransfected N2A cell membranes that endogenously expressed CB_1_R, N2A cell membranes co-expressing CB_1_R with either DOR, MOR, KOR, CB_2_R or AT1R and HEK293 membranes expressing either DOR, KOR and DOR, or MOR and DOR . Cell lines were screened using 1∶10 hybridoma supernatant and 1∶500 horseradish peroxidase labeled anti-mouse IgG. Only hybridoma supernatants from clones that gave a specific signal with CB_1_R-DOR were further purified as described [82] and screened for specificity against cortical membranes prepared from wild-type, CB_1_R −/− and DOR −/− animals.

### ELISA

Quantitation of levels of receptors following peripheral lesion was carried out by ELISA. Membranes (from n = 7 animals per group) were diluted in PBS (5 µg/100 µL) and coated onto 96-well plates (5 µg/well). All experiments were carried out in triplicate. Membranes were fixed with 4% paraformaldehyde (PFA) in phosphate buffered saline (PBS) for 20 min and then washed five times in PBS, followed by incubation in blocking buffer (1% bovine serum albumin (BSA) in PBS) for 90 min. Membranes were incubated in primary antibody overnight at 4°C. Primary antibodies used included rabbit polyclonal antibody against C-term CB_1_R (Cayman Chemical, Ann Arbor, MI, USA) (1∶300), a rat polyclonal antibody directed against DOR ([68]) (1∶1000) and a mouse monoclonal antibody directed against CB_1_R-DOR (1∶100). After washing in PBS, horse radish peroxidase conjugated secondary antibody was added for 90 min. After final washes in PBS, colorimetric substrate was added and the plate was scanned at 490 nm.

### Quantitative Real-Time RT-PCR

The relative levels of receptor mRNA were quantified by quantitative PCR [26], [91]. Total RNA was isolated from various brain regions using the TRIzol method (Invitrogen, Carlsbad, CA, USA). RNA (1.0 µg) was reverse transcribed in 20 µLof buffer containing 50 µM oligo(dT)_20_, 25 mM MgCl_2_, 0.1 M dithiothreitol, 40 U/µL RNaseOUT, and 200 U/µL SuperScript III RT for 50 min at 50°C. The reaction was stopped by incubating the samples at 85°C for 5 min, after which 40 µl of nuclease free water was added. Real-time PCR was performed by using the Brilliant SYBR Green QPCR Master Mix (Agilent Technologies, Santa Clara, CA, USA). The PCR template source was either 30 ng of first-strand cDNA or purified DNA standard. Primers used were: (i) CTTCCGTACCATCACCACAG (forward) and GAAGGGACTACCCCTGAAGG (reverse) or (ii) TGTCTCCCATTTCAAGCAAG and GGTGATGGTACGGAAGGTG (reverse). Amplification was performed with a spectrofluorometric thermal cycler (Stratagene, La Jolla, CA, USA). After an initial denaturation step at 95°C for 10 min, amplification was performed using 40 cycles of denaturation (95°C for 30 s), annealing (56°C for 1 min), and extension (72°C for 1 min). To standardize mRNA levels, GAPDH, a housekeeping gene, was used as an internal control. Gene expression was normalized by calculating the ratio between the number of cDNA copies of CB_1_R and that of GAPDH in both sham and lesion conditions.

### Immunohistochemistry

Localization of CB_1_R within the cortex of sham and lesioned rats, 3, 7 and 14 days after surgery, was evaluated by immunohistochemistry. Rats were deeply anesthetized with 100 mg/kg chloral hydrate and perfused transcardially with 0.1 M PBS followed by 4% PFA in PBS. Tissues were dissected, post-fixed in 4% PFA in PBS for 4 h, and cryoprotected overnight in 30% sucrose in PBS. Brains were sectioned on a Leica VT 1000S vibratome (Leica Biosystems, Buffalo Grove, IL, USA) at 50 µm and processed as free-floating sections. Tissue was incubated for 1 h in a blocking solution containing 0.1 M PBS with 0.3% Triton X-100 plus 5% normal donkey serum (Jackson Immunoresearch, West Grove, PA, USA). Primary and secondary antibodies were diluted in PBS containing 0.3% Triton X-100 plus 1% normal donkey serum. CB_1_R was labeled with a rabbit polyclonal primary antibody directed against the C-terminus of CB_1_R (Cayman Chemical, Ann Arbor, MI, USA) (1∶5000), and was visualized with an Alexa goat-anti-rabbit 594 secondary antibody (Invitrogen, Grand Island, NY, USA) (1∶1000). Tissue sections were incubated overnight at 4°C in primary antibody, washed in PBS and then incubated for a further 2 h in secondary antibody at RT. Images were acquired with a Zeiss LSM510 Meta confocal microscope (Carl Zeiss, Thornwood, NY, USA). Typical sampling for this analysis was ∼4 microscope fields (acquired at 1024×1024 pixel resolution, with a z-step of 0.1 µm) and ∼2 tissue sections equally spaced through the cortical layer of interest. For each sample, average intensity values were determined using ImageJ (NIH) software.

### [^35^S]GTPγS Binding

Peripheral nerve lesion-induced changes in receptor activity were measured using [^35^S]GTPγS binding. Briefly, membranes (n = 6–7 animals per group) from sham or lesioned animals (14 days post-surgery) were incubated with increasing concentrations of Hu-210 (0.1 pM to 10 µM) or DPDPE (1 pM to 10 µM) in the presence of 2 mM GDP and 0.5 nM [^35^S]GTPγS as described in [94]–[96]. Basal binding in the presence of GDP and an absence of agonist and cold GTPγS was also determined. Non-specific binding was determined by the addition of 10 µM cold GTPγS to a parallel set of tubes. The radioactivity bound to membranes was separated by filtration and quantified by scintillation counting. Dose dependent activation of [^35^S]GTPγS binding by DPDPE was also measured in the presence of a non-activating concentration of Hu-210 (1 pM), or PF-514273 (1 µM) in cortical membranes from sham or lesioned animals. [^35^S]GTPγS binding was analyzed by calculating EC_50_ and E_max_ values for each set of experiments. Activation of [^35^S]GTPγS binding by 10 µM DPDPE±1 pM Hu-210 was also measured in the presence of a non activating concentration of DAMGO (10 nM) or U69593 (10 nM) or in the presence of 1 µg of the following antibodies (CB_1_R-DOR mAb, CB_1_R-AT1R mAb [26], MOR-DOR mAb [25], CB_1_R Ab, DOR Ab or non-specific IgG (Santa Cruz Biotechnology, Inc., Santa Cruz, CA, USA) in cortical membranes from lesioned animals.

### Radioligand Binding

Membranes were prepared from cortices of sham and lesioned rats, as well as from N2A cells stably expressing DOR [22] or N2A-DOR cells in which CB_1_R expression was knocked down by siRNA transfection (pooled siRNAs against CB_1_R; from Santa Cruz Biotechnology, Inc., Santa Cruz, CA, USA). For all ligand binding experiments, membranes were added to cold assay buffer containing 50 mM Tris, 1 mg/ml fatty acid-free BSA, 10 mM MgCl_2_ and 0.5 mM DTT. Non-specific binding was assessed using 10 µM DPDPE. Total binding was measured using 0.5 nM [^3^H]DPDPE in the absence or presence of indicated concentrations of Hu-210 or PF-514273, in the absence or presence of 1 µg of CB_1_R-DOR monoclonal antibody (mAb). Binding assays were carried out for 120 min at 30°C. Membranes were filtered and radioactivity was measured using a liquid scintillation counter.

### Statistcal Methods and Analysis

For all experiments, changes in group differences were evaluated by using a repeated measures ANOVA followed by a Student's post-hoc test. A *p*-value of <0.05 was considered to be statistically significant for all tests. All statistical operations were performed using GraphPad Prism, Version 5.0 (GraphPad Software, La Jolla, CA, USA). For the analysis of [^35^S]GTPγS binding results, values from each experimental condition (e.g. DPDPE dose response curve ±1 pM Hu-210) were compared using a one-way ANOVA. Here again, a *p*-value<0.05 was considered to be statistically significant. The reported r^2^ value was calculated from linear correlation analysis.

## References

[1] Finnerup NB, Sindrup SH, Jensen TS (2010) The evidence for pharmacological treatment of neuropathic pain. Pain 150: 573–581. 20705215 10.1016/j.pain.2010.06.019

[2] O'Connor AB, Dworkin RH (2009) Treatment of neuropathic pain: an overview of recent guidelines. Am J Med 122: S22–32. 10.1016/j.amjmed.2009.04.007 19801049

[3] Bausch SB, Patterson TA, Appleyard SM, Chavkin C (1995) Immunocytochemical localization of delta opioid receptors in mouse brain. J Chem Neuroanat 8: 175–189. 7598816 10.1016/0891-0618(94)00044-t

[4] Hohmann AG, Briley EM, Herkenham M (1999) Pre- and postsynaptic distribution of cannabinoid and mu opioid receptors in rat spinal cord. Brain Res 822: 17–25. 10082879 10.1016/s0006-8993(98)01321-3

[5] Maldonado R, Valverde O (2003) Participation of the opioid system in cannabinoid-induced antinociception and emotional-like responses. Eur Neuropsychopharmacol 13: 401–410. 14636956 10.1016/j.euroneuro.2003.08.001

[6] Manzanares J, Corchero J, Romero J, Fernandez-Ruiz JJ, Ramos JA, et al (1999) Pharmacological and biochemical interactions between opioids and cannabinoids. Trends Pharmacol Sci 20: 287–294. 10390647 10.1016/s0165-6147(99)01339-5

[7] Pan HL, Wu ZZ, Zhou HY, Chen SR, Zhang HM, et al (2008) Modulation of pain transmission by G-protein-coupled receptors. Pharmacol Ther 117: 141–161. 17959251 10.1016/j.pharmthera.2007.09.003 PMC2965406

[8] Salio C, Fischer J, Franzoni MF, Conrath M (2002) Pre- and postsynaptic localizations of the CB1 cannabinoid receptor in the dorsal horn of the rat spinal cord. Neuroscience 110: 755–764. 11934482 10.1016/s0306-4522(01)00584-x

[9] Svizenska I, Dubovy P, Sulcova A (2008) Cannabinoid receptors 1 and 2 (CB1 and CB2), their distribution, ligands and functional involvement in nervous system structures–a short review. Pharmacol Biochem Behav 90: 501–511. 18584858 10.1016/j.pbb.2008.05.010

[10] Wilson-Poe AR, Morgan MM, Aicher SA, Hegarty DM (2012) Distribution of CB1 cannabinoid receptors and their relationship with mu-opioid receptors in the rat periaqueductal gray. Neuroscience 213: 191–200. 22521830 10.1016/j.neuroscience.2012.03.038 PMC3367071

[11] Bie B, Pan ZZ (2007) Trafficking of central opioid receptors and descending pain inhibition. Mol Pain 3: 37. 18053223 10.1186/1744-8069-3-37 PMC2219988

[12] Pertwee RG (2001) Cannabinoid receptors and pain. Prog Neurobiol 63: 569–611. 11164622 10.1016/s0301-0082(00)00031-9

[13] Bushlin I, Rozenfeld R, Devi LA (2010) Cannabinoid-opioid interactions during neuropathic pain and analgesia. Curr Opin Pharmacol 10: 80–86. 19857996 10.1016/j.coph.2009.09.009 PMC2818338

[14] Welch SP (2009) Interaction of the cannabinoid and opioid systems in the modulation of nociception. Int Rev Psychiatry 21: 143–151. 19367508 10.1080/09540260902782794

[15] Parolaro D, Rubino T, Vigano D, Massi P, Guidali C, et al (2010) Cellular mechanisms underlying the interaction between cannabinoid and opioid system. Curr Drug Targets 11: 393–405. 20017730 10.2174/138945010790980367

[16] Perrine SA, Hoshaw BA, Unterwald EM (2006) Delta opioid receptor ligands modulate anxiety-like behaviors in the rat. Br J Pharmacol 147: 864–872. 16491101 10.1038/sj.bjp.0706686 PMC1760715

[17] Saitoh A, Kimura Y, Suzuki T, Kawai K, Nagase H, et al (2004) Potential anxiolytic and antidepressant-like activities of SNC80, a selective delta-opioid agonist, in behavioral models in rodents. J Pharmacol Sci 95: 374–380. 15272214 10.1254/jphs.fpj04014x

[18] Bambico FR, Katz N, Debonnel G, Gobbi G (2007) Cannabinoids elicit antidepressant-like behavior and activate serotonergic neurons through the medial prefrontal cortex. J Neurosci 27: 11700–11711. 17959812 10.1523/JNEUROSCI.1636-07.2007 PMC6673235

[19] Lin HC, Mao SC, Su CL, Gean PW (2009) The role of prefrontal cortex CB1 receptors in the modulation of fear memory. Cereb Cortex 19: 165–175. 18477688 10.1093/cercor/bhn075

[20] Rea K, Roche M, Finn DP (2007) Supraspinal modulation of pain by cannabinoids: the role of GABA and glutamate. Br J Pharmacol 152: 633–648. 17828292 10.1038/sj.bjp.0707440 PMC2190023

[21] Uriguen L, Berrendero F, Ledent C, Maldonado R, Manzanares J (2005) Kappa- and delta-opioid receptor functional activities are increased in the caudate putamen of cannabinoid CB1 receptor knockout mice. Eur J Neurosci 22: 2106–2110. 16262648 10.1111/j.1460-9568.2005.04372.x

[22] Rozenfeld R, Bushlin I, Gomes I, Tzavaras N, Gupta A, et al (2012) Receptor heteromerization expands the repertoire of cannabinoid signaling in rodent neurons. PLoS One 7: e29239. 22235275 10.1371/journal.pone.0029239 PMC3250422

[23] Berrendero F, Mendizabal V, Murtra P, Kieffer BL, Maldonado R (2003) Cannabinoid receptor and WIN 55 212–2-stimulated [35S]-GTPgammaS binding in the brain of mu-, delta- and kappa-opioid receptor knockout mice. Eur J Neurosci 18: 2197–2202. 14622180 10.1046/j.1460-9568.2003.02951.x

[24] Berrendero F, Maldonado R (2002) Involvement of the opioid system in the anxiolytic-like effects induced by Delta(9)-tetrahydrocannabinol. Psychopharmacology (Berl) 163: 111–117. 12185408 10.1007/s00213-002-1144-9

[25] Gupta A, Mulder J, Gomes I, Rozenfeld R, Bushlin I, et al (2010) Increased abundance of opioid receptor heteromers after chronic morphine administration. Sci Signal 3: ra54. 20647592 10.1126/scisignal.2000807 PMC3125674

[26] Rozenfeld R, Gupta A, Gagnidze K, Lim MP, Gomes I, et al (2011) AT1R-CB(1)R heteromerization reveals a new mechanism for the pathogenic properties of angiotensin II. Embo J 30: 2350–2363. 21540834 10.1038/emboj.2011.139 PMC3116274

[27] Rios C, Gomes I, Devi LA (2006) mu opioid and CB1 cannabinoid receptor interactions: reciprocal inhibition of receptor signaling and neuritogenesis. Br J Pharmacol 148: 387–395. 16682964 10.1038/sj.bjp.0706757 PMC1751792

[28] Ji RR, Zhang Q, Law PY, Low HH, Elde R, et al (1995) Expression of mu-, delta-, and kappa-opioid receptor-like immunoreactivities in rat dorsal root ganglia after carrageenan-induced inflammation. J Neurosci 15: 8156–8166. 8613750 10.1523/JNEUROSCI.15-12-08156.1995 PMC6577939

[29] Kabli N, Cahill CM (2007) Anti-allodynic effects of peripheral delta opioid receptors in neuropathic pain. Pain 127: 84–93. 16963185 10.1016/j.pain.2006.08.003

[30] Li JL, Kaneko T, Mizuno N (1996) Effects of peripheral nerve ligation on expression of mu-opioid receptor in sensory ganglion neurons: an immunohistochemical study in dorsal root and nodose ganglion neurons of the rat. Neurosci Lett 214: 91–94. 8878091 10.1016/0304-3940(96)12894-9

[31] Puehler W, Zollner C, Brack A, Shaqura MA, Krause H, et al (2004) Rapid upregulation of mu opioid receptor mRNA in dorsal root ganglia in response to peripheral inflammation depends on neuronal conduction. Neuroscience 129: 473–479. 15501604 10.1016/j.neuroscience.2004.06.086

[32] Truong W, Cheng C, Xu QG, Li XQ, Zochodne DW (2003) Mu opioid receptors and analgesia at the site of a peripheral nerve injury. Ann Neurol 53: 366–375. 12601704 10.1002/ana.10465

[33] Walczak JS, Pichette V, Leblond F, Desbiens K, Beaulieu P (2005) Behavioral, pharmacological and molecular characterization of the saphenous nerve partial ligation: a new model of neuropathic pain. Neuroscience 132: 1093–1102. 15857713 10.1016/j.neuroscience.2005.02.010

[34] Obara I, Parkitna JR, Korostynski M, Makuch W, Kaminska D, et al (2009) Local peripheral opioid effects and expression of opioid genes in the spinal cord and dorsal root ganglia in neuropathic and inflammatory pain. Pain 141: 283–291. 19147290 10.1016/j.pain.2008.12.006

[35] Rashid MH, Inoue M, Toda K, Ueda H (2004) Loss of peripheral morphine analgesia contributes to the reduced effectiveness of systemic morphine in neuropathic pain. J Pharmacol Exp Ther 309: 380–387. 14718584 10.1124/jpet.103.060582

[36] Zhang J, Ferguson SS, Barak LS, Bodduluri SR, Laporte SA, et al (1998) Role for G protein-coupled receptor kinase in agonist-specific regulation of mu-opioid receptor responsiveness. Proc Natl Acad Sci U S A 95: 7157–7162. 9618555 10.1073/pnas.95.12.7157 PMC22772

[37] Besse D, Lombard MC, Perrot S, Besson JM (1992) Regulation of opioid binding sites in the superficial dorsal horn of the rat spinal cord following loose ligation of the sciatic nerve: comparison with sciatic nerve section and lumbar dorsal rhizotomy. Neuroscience 50: 921–933. 1333063 10.1016/0306-4522(92)90215-n

[38] Stevens CW, Kajander KC, Bennett GJ, Seybold VS (1991) Bilateral and differential changes in spinal mu, delta and kappa opioid binding in rats with a painful, unilateral neuropathy. Pain 46: 315–326. 1661886 10.1016/0304-3959(91)90114-D

[39] Datta S, Chatterjee K, Kline RHt, Wiley RG (2010) Behavioral and anatomical characterization of the bilateral sciatic nerve chronic constriction (bCCI) injury: correlation of anatomic changes and responses to cold stimuli. Mol Pain 6: 7. 20105332 10.1186/1744-8069-6-7 PMC2825192

[40] Holdridge SV, Cahill CM (2007) Spinal administration of a delta opioid receptor agonist attenuates hyperalgesia and allodynia in a rat model of neuropathic pain. Eur J Pain 11: 685–693. 17175187 10.1016/j.ejpain.2006.10.008

[41] Porreca F, Tang QB, Bian D, Riedl M, Elde R, et al (1998) Spinal opioid mu receptor expression in lumbar spinal cord of rats following nerve injury. Brain Res 795: 197–203. 9622629 10.1016/s0006-8993(98)00292-3

[42] Robertson B, Schulte G, Elde R, Grant G (1999) Effects of sciatic nerve injuries on delta -opioid receptor and substance P immunoreactivities in the superficial dorsal horn of the rat. Eur J Pain 3: 115–129. 10700341 10.1053/eujp.1998.0104

[43] Stone LS, Vulchanova L, Riedl MS, Williams FG, Wilcox GL, et al (2004) Effects of peripheral nerve injury on delta opioid receptor (DOR) immunoreactivity in the rat spinal cord. Neurosci Lett 361: 208–211. 15135930 10.1016/j.neulet.2003.12.067

[44] Tseng TJ, Chen CC, Hsieh YL, Hsieh ST (2008) Influences of surgical decompression on the dorsal horn after chronic constriction injury: changes in peptidergic and delta-opioid receptor (+) nerve terminals. Neuroscience 156: 758–768. 18773941 10.1016/j.neuroscience.2008.08.010

[45] Wang Z, Gardell LR, Ossipov MH, Vanderah TW, Brennan MB, et al (2001) Pronociceptive actions of dynorphin maintain chronic neuropathic pain. J Neurosci 21: 1779–1786. 11222667 10.1523/JNEUROSCI.21-05-01779.2001 PMC6762963

[46] Lim G, Sung B, Ji RR, Mao J (2003) Upregulation of spinal cannabinoid-1-receptors following nerve injury enhances the effects of Win 55,212-2 on neuropathic pain behaviors in rats. Pain 105: 275–283. 14499445 10.1016/s0304-3959(03)00242-2

[47] Mitrirattanakul S, Ramakul N, Guerrero AV, Matsuka Y, Ono T, et al (2006) Site-specific increases in peripheral cannabinoid receptors and their endogenous ligands in a model of neuropathic pain. Pain 126: 102–114. 16844297 10.1016/j.pain.2006.06.016 PMC1776167

[48] Wang S, Lim G, Mao J, Sung B, Yang L, et al (2007) Central glucocorticoid receptors regulate the upregulation of spinal cannabinoid-1 receptors after peripheral nerve injury in rats. Pain 131: 96–105. 17258396 10.1016/j.pain.2006.12.019

[49] Paszcuk AF, Dutra RC, da Silva KA, Quintao NL, Campos MM, et al (2011) Cannabinoid agonists inhibit neuropathic pain induced by brachial plexus avulsion in mice by affecting glial cells and MAP kinases. PLoS One 6: e24034. 21931637 10.1371/journal.pone.0024034 PMC3172222

[50] Pertwee RG (2009) Emerging strategies for exploiting cannabinoid receptor agonists as medicines. Br J Pharmacol 156: 397–411. 19226257 10.1111/j.1476-5381.2008.00048.x PMC2697681

[51] Saade NE, Jabbur SJ (2008) Nociceptive behavior in animal models for peripheral neuropathy: spinal and supraspinal mechanisms. Prog Neurobiol 86: 22–47. 18602968 10.1016/j.pneurobio.2008.06.002

[52] Seifert F, Maihofner C (2009) Central mechanisms of experimental and chronic neuropathic pain: findings from functional imaging studies. Cell Mol Life Sci 66: 375–390. 18791842 10.1007/s00018-008-8428-0 PMC11131450

[53] Jutkiewicz EM, Torregrossa MM, Sobczyk-Kojiro K, Mosberg HI, Folk JE, et al (2006) Behavioral and neurobiological effects of the enkephalinase inhibitor RB101 relative to its antidepressant effects. Eur J Pharmacol 531: 151–159. 16442521 10.1016/j.ejphar.2005.12.002 PMC1828120

[54] Saitoh A, Sugiyama A, Nemoto T, Fujii H, Wada K, et al (2011) The novel delta opioid receptor agonist KNT-127 produces antidepressant-like and antinociceptive effects in mice without producing convulsions. Behav Brain Res 223: 271–279. 21565223 10.1016/j.bbr.2011.04.041

[55] Narita M, Kuzumaki N, Kaneko C, Hareyama N, Miyatake M, et al (2006) Chronic pain-induced emotional dysfunction is associated with astrogliosis due to cortical delta-opioid receptor dysfunction. J Neurochem 97: 1369–1378. 16696849 10.1111/j.1471-4159.2006.03824.x

[56] Saitoh A, Yoshikawa Y, Onodera K, Kamei J (2005) Role of delta-opioid receptor subtypes in anxiety-related behaviors in the elevated plus-maze in rats. Psychopharmacology (Berl) 182: 327–334. 16075288 10.1007/s00213-005-0112-6

[57] Filliol D, Ghozland S, Chluba J, Martin M, Matthes HW, et al (2000) Mice deficient for delta- and mu-opioid receptors exhibit opposing alterations of emotional responses. Nat Genet 25: 195–200. 10835636 10.1038/76061

[58] Rodriguez de Fonseca F, Cebeira M, Ramos JA, Martin M, Fernandez-Ruiz JJ (1994) Cannabinoid receptors in rat brain areas: sexual differences, fluctuations during estrous cycle and changes after gonadectomy and sex steroid replacement. Life Sci 54: 159–170. 8289577 10.1016/0024-3205(94)00585-0

[59] Kathuria S, Gaetani S, Fegley D, Valino F, Duranti A, et al (2003) Modulation of anxiety through blockade of anandamide hydrolysis. Nat Med 9: 76–81. 12461523 10.1038/nm803

[60] Patel S, Hillard CJ (2006) Pharmacological evaluation of cannabinoid receptor ligands in a mouse model of anxiety: further evidence for an anxiolytic role for endogenous cannabinoid signaling. J Pharmacol Exp Ther 318: 304–311. 16569753 10.1124/jpet.106.101287

[61] Ozaki S, Narita M, Narita M, Iino M, Miyoshi K, et al (2003) Suppression of the morphine-induced rewarding effect and G-protein activation in the lower midbrain following nerve injury in the mouse: involvement of G-protein-coupled receptor kinase 2. Neuroscience 116: 89–97. 12535942 10.1016/s0306-4522(02)00699-1

[62] Hoot MR, Sim-Selley LJ, Poklis JL, Abdullah RA, Scoggins KL, et al (2010) Chronic constriction injury reduces cannabinoid receptor 1 activity in the rostral anterior cingulate cortex of mice. Brain Res 1339: 18–25. 20380816 10.1016/j.brainres.2010.03.105 PMC3736380

[63] Knerlich-Lukoschus F, Noack M, von der Ropp-Brenner B, Lucius R, Mehdorn HM, et al (2011) Spinal Cord Injuries Induce Changes in CB(1) Cannabinoid Receptor and C-C Chemokine Expression in Brain Areas Underlying Circuitry of Chronic Pain Conditions. J Neurotrauma 28: 619–634. 21265596 10.1089/neu.2010.1652

[64] Chung JM, Kim HK, Chung K (2004) Segmental spinal nerve ligation model of neuropathic pain. Methods Mol Med 99: 35–45. 15131327 10.1385/1-59259-770-X:035

[65] Kim SH, Chung JM (1992) An experimental model for peripheral neuropathy produced by segmental spinal nerve ligation in the rat. Pain 50: 355–363. 1333581 10.1016/0304-3959(92)90041-9

[66] Beltramo M, Bernardini N, Bertorelli R, Campanella M, Nicolussi E, et al (2006) CB2 receptor-mediated antihyperalgesia: possible direct involvement of neural mechanisms. Eur J Neurosci 23: 1530–1538. 16553616 10.1111/j.1460-9568.2006.04684.x

[67] Jhaveri MD, Richardson D, Kendall DA, Barrett DA, Chapman V (2006) Analgesic effects of fatty acid amide hydrolase inhibition in a rat model of neuropathic pain. J Neurosci 26: 13318–13327. 17182782 10.1523/JNEUROSCI.3326-06.2006 PMC6674985

[68] Scott DA, Wright CE, Angus JA (2004) Evidence that CB-1 and CB-2 cannabinoid receptors mediate antinociception in neuropathic pain in the rat. Pain 109: 124–131. 15082134 10.1016/j.pain.2004.01.020

[69] Liu CN, Wall PD, Ben-Dor E, Michaelis M, Amir R, et al (2000) Tactile allodynia in the absence of C-fiber activation: altered firing properties of DRG neurons following spinal nerve injury. Pain 85: 503–521. 10781925 10.1016/S0304-3959(00)00251-7

[70] Wu G, Ringkamp M, Hartke TV, Murinson BB, Campbell JN, et al (2001) Early onset of spontaneous activity in uninjured C-fiber nociceptors after injury to neighboring nerve fibers. J Neurosci 21: RC140. 11306646 10.1523/JNEUROSCI.21-08-j0002.2001 PMC6762537

[71] Suzuki T, Amata M, Sakaue G, Nishimura S, Inoue T, et al (2007) Experimental neuropathy in mice is associated with delayed behavioral changes related to anxiety and depression. Anesth Analg 104: 1570–1577, table of contents. 17513660 10.1213/01.ane.0000261514.19946.66

[72] Hungund BL, Vinod KY, Kassir SA, Basavarajappa BS, Yalamanchili R, et al (2004) Upregulation of CB1 receptors and agonist-stimulated [35S]GTPgammaS binding in the prefrontal cortex of depressed suicide victims. Mol Psychiatry 9: 184–190. 14966476 10.1038/sj.mp.4001376

[73] Vinod KY, Arango V, Xie S, Kassir SA, Mann JJ, et al (2005) Elevated levels of endocannabinoids and CB1 receptor-mediated G-protein signaling in the prefrontal cortex of alcoholic suicide victims. Biol Psychiatry 57: 480–486. 15737662 10.1016/j.biopsych.2004.11.033

[74] Dubois D, Gendron L (2010) Delta opioid receptor-mediated analgesia is not altered in preprotachykinin A knockout mice. Eur J Neurosci 32: 1921–1929. 21044181 10.1111/j.1460-9568.2010.07466.x

[75] Mackie K (2005) Distribution of cannabinoid receptors in the central and peripheral nervous system. Handb Exp Pharmacol 168: 299–325. 10.1007/3-540-26573-2_10 16596779

[76] Pertwee RG (2005) The therapeutic potential of drugs that target cannabinoid receptors or modulate the tissue levels or actions of endocannabinoids. AAPS J 7: E625–654. 16353941 10.1208/aapsj070364 PMC2751266

[77] Springael JY, Urizar E, Costagliola S, Vassart G, Parmentier M (2007) Allosteric properties of G protein-coupled receptor oligomers. Pharmacol. Ther 115: 410–418. 10.1016/j.pharmthera.2007.06.004 17655934

[78] Kathmann M, Flau K, Redmer A, Trankle C, Schlicker E (2006) Cannabidiol is an allosteric modulator at mu- and delta-opioid receptors. Naunyn Schmiedebergs Arch Pharmacol 372: 354–361. 16489449 10.1007/s00210-006-0033-x

[79] Gomes I, Ijzerman AP, Ye K, Maillet EL, Devi LA (2011) G protein-coupled receptor heterodimerization: A role in allosteric modulation of ligand-mediated receptor binding. Mol Pharmacol 9: 1044–52. 10.1124/mol.110.070847 PMC3102551 21415307

[80] Rozenfeld R, Devi LA (2010) Receptor heteromerization and drug discovery. Trends Pharmacol Sci 31: 124–130. 20060175 10.1016/j.tips.2009.11.008 PMC2834828

[81] Ferré S, Navarro G, Casadó V, Cortés A, Mallol J, Canela EI, Lluís C, Franco R (2010) G protein-coupled receptor heteromers as new targets for drug development. Prog Mol Biol Transl Sci 91: 41–52. 20691958 10.1016/S1877-1173(10)91002-8 PMC9361225

[82] Gomes I, Gupta A, Devi LA (2012) G protein-coupled receptor heteromer regualtion in disease. Methods in Enzymology (in press) 10.1016/B978-0-12-391862-8.00012-0 PMC3976561 23351742

[83] Abul-Husn NS, Sutak M, Milne B, Jhamandas K (2007) Augmentation of spinal morphine analgesia and inhibition of tolerance by low doses of mu- and delta-opioid receptor antagonists. Br J Pharmacol 151: 877–887. 17502848 10.1038/sj.bjp.0707277 PMC2014123

[84] Gomes I, Gupta A, Filipovska J, Szeto HH, Pintar JE, et al (2004) A role for heterodimerization of mu and delta opiate receptors in enhancing morphine analgesia. Proc Natl Acad Sci U S A 101: 5135–5139. 15044695 10.1073/pnas.0307601101 PMC387386

[85] Daniels DJ, Lenard NR, Etienne CL, Law PY, Roerig SC, et al (2005) Opioid-induced tolerance and dependence in mice is modulated by the distance between pharmacophores in a bivalent ligand series. Proc Natl Acad Sci U S A 102: 19208–19213. 16365317 10.1073/pnas.0506627102 PMC1323165

[86] He SQ, Zhang ZN, Guan JS, Liu HR, Zhao B, et al (2011) Facilitation of mu-opioid receptor activity by preventing delta-opioid receptor-mediated codegradation. Neuron 69: 120–131. 21220103 10.1016/j.neuron.2010.12.001

[87] Pei L, Li S, Wang M, Diwan M, Anisman H, et al (2010) Uncoupling the dopamine D1–D2 receptor complex exerts antidepressant-like effects. Nat Med 16: 1393–1395. 21113156 10.1038/nm.2263

[88] Berg KA, Rowan MP, Gupta A, Sanchez TA, Silva M, Gomes I, McGuire BA, Portoghese PS, Hargreaves KM, Devi LA, Clarke WP (2012) Allosteric Interactions between Delta and Kappa Opioid Receptors in Peripheral Sensory Neurons. Mol. Pharmacol 81: 264–72. 22072818 10.1124/mol.111.072702 PMC3263945

[89] Chaplan SR, Bach FW, Pogrel JW, Chung JM, Yaksh TL (1994) Quantitative assessment of tactile allodynia in the rat paw. J Neurosci Methods 53: 55–63. 7990513 10.1016/0165-0270(94)90144-9

[90] Dixon WJ (1980) Efficient analysis of experimental observations. Annu Rev Pharmacol Toxicol 20: 441–462. 7387124 10.1146/annurev.pa.20.040180.002301

[91] Lim MP, Devi LA, Rozenfeld R (2011) Cannabidiol causes activated hepatic stellate cell death through a mechanism of endoplasmic reticulum stress-induced apoptosis. Cell Death Dis 9: e170. 10.1038/cddis.2011.52 PMC3168994 21654828

[92] Takahashi KA, Castillo PE (2006) The CB1 cannabinoid receptor mediates glutamatergic synaptic suppression in the hippocampus. Neuroscience 139: 795–802. 16527424 10.1016/j.neuroscience.2006.01.024

[93] Gupta A, Decaillot FM, Gomes I, Tkalych O, Heimann AS, et al (2007) Conformation state-sensitive antibodies to G-protein-coupled receptors. J Biol Chem 282: 5116–5124. 17148456 10.1074/jbc.M609254200 PMC3856726

[94] Gomes I, Jordan BA, Gupta A, Trapaidze N, Nagy V, et al (2000) Heterodimerization of mu and delta opioid receptors: A role in opiate synergy. J Neurosci 20: RC110. 11069979 10.1523/JNEUROSCI.20-22-j0007.2000 PMC3125672

[95] Harrison C, Traynor JR (2003) The [35S]GTPgammaS binding assay: approaches and applications in pharmacology. Life Sci 74: 489–508. 14609727 10.1016/j.lfs.2003.07.005

[96] Lazareno S (1999) Measurement of agonist-stimulated [35S]GTP gamma S binding to cell membranes. Methods Mol Biol 106: 231–245. 9921508 10.1385/0-89603-530-1:231

